# Rumen Protozoa Play a Significant Role in Fungal Predation and Plant Carbohydrate Breakdown

**DOI:** 10.3389/fmicb.2020.00720

**Published:** 2020-04-29

**Authors:** Cate L. Williams, Benjamin J. Thomas, Neil R. McEwan, Pauline Rees Stevens, Christopher J. Creevey, Sharon A. Huws

**Affiliations:** ^1^Institute of Biological, Environmental and Rural Science, Aberystwyth University, Aberystwyth, United Kingdom; ^2^School of Biological Sciences, Institute for Global Food Security, Queen’s University Belfast, Belfast, United Kingdom; ^3^School of Pharmacy and Life Sciences, Robert Gordon University, Aberdeen, United Kingdom

**Keywords:** rumen, protozoa, carbohydrate metabolism, metatranscriptome, eukaryotes, metagenomic library

## Abstract

The rumen protozoa, alongside fungi, comprise the eukaryotic portion of the rumen microbiome. Rumen protozoa may account for up to 50% of biomass, yet their role in this ecosystem remains unclear. Early experiments inferred a role in carbohydrate and protein metabolism, but due to their close association with bacteria, definitively attributing these functions to the protozoa was challenging. The advent of ‘omic technologies has created opportunities to broaden our understanding of the rumen protozoa. This study aimed to utilize these methods to further our understanding of the role that protozoa play in the rumen in terms of their metabolic capacities, and in doing so, contribute valuable sequence data to reduce the chance of mis or under-representation of the rumen protozoa in meta’omic datasets. Rumen protozoa were isolated and purified using glucose-based sedimentation and differential centrifugation, extracted RNA was Poly(A) fraction enriched and DNase treated before use in a phage-based, cDNA metatranscriptomic library. Biochemical activity testing of the phage library showed 6 putatively positive plaques in response to carboxymethyl cellulose agar (indicative of cellulose activity), and no positive results for tributyrin (indicative of esterase/lipase activity) or egg yolk agar (indicative of proteolysis). Direct sequencing of the cDNA was also conducted using the Illumina HiSeq 2500. The metatranscriptome identified a wealth of carbohydrate-active enzymes which accounted for 8% of total reads. The most highly expressed carbohydrate-active enzymes were glycosyl hydrolases 5 and 11, polysaccharide lyases and deacetylases, xylanases and enzymes active against pectin, mannan and chitin; the latter likely used to digest rumen fungi which contain a chitin-rich cell membrane. Codon usage analysis of expressed genes also showed evidence of horizontal gene transfer, suggesting that many of these enzymes were acquired from the rumen bacteria in an evolutionary response to the carbohydrate-rich environment of the rumen. This study provides evidence of the significant contribution that the protozoa make to carbohydrate breakdown in the rumen, potentially using horizontally acquired genes, and highlights their predatory capacity.

## Introduction

Rumen protozoa are classified into 2 groups, namely the entodinomorphs and holotrichs. These protozoa are distinctive in terms of their phenotypic and behavioral adaptations, which allow survival in this harsh anaerobic environment. The rumen is warm (38.4–39.8°C), highly anoxic and contracts frequently to increase the flow of particles through the subsequent chambers of the foregut and into the lower intestines (García et al., 2018). The slow generation time of the protozoa which ranges from 6 to 55 h (Karnati et al., 2007; Sylvester et al., 2009; Denton et al., 2015) has been circumvented by a behavioral adaptation largely resulting in finding niches to hide in during contraction, such as the epithelial wall (Abe et al., 1981).

The rumen protozoa are well known for their fibrolytic activity; however, most enzymatic activity has only been inferred, either by defaunation experiments or in mixed *in vitro* experiments with bacteria or fungi (Forsberg et al., 1984; Orpin, 1984; Varel and Dehority, 1989; Jouany and Ushida, 1999). As such, very few protozoal enzymes have been identified and characterized. The first, and only, draft macronuclear genome sequence of rumen protozoa (*Entodinium caudatum*) was only recently published (Park et al., 2018). In addition to a high A-T bias, rumen protozoa are further complicated by the presence of two types of nuclei: the macro and micronucleus. This lack of reference sequences for rumen protozoa often results in their mis- or under-representation in meta ‘omic datasets as well as poor coverage during annotation (Comtet-Marre et al., 2017). Nonetheless, recent developments in the field of ‘omics, allow researchers to delve further than ever before into otherwise challenging microbiomes.

Acquisition of functionally important genes via horizontal gene transfer (HGT), from bacteria into protozoa, has also been demonstrated (Ricard et al., 2006). In particular, many genes encoding enzymes involved in carbohydrate transfer and metabolism appeared to have been acquired through HGT from bacteria (<75%), with 35% of these being glycosyl hydrolases, Interestingly, a distinct difference was observed between the enzyme profiles of entodiniomorphids and holotrichs, supporting the hypothesis that these protozoal groups occupy a slightly different niche in the rumen environment and possess varying functionality in terms of rumen metabolism. Ricard et al. (2006) suggests that HGT is an important process in the adaptation of rumen protozoa to a carbohydrate-rich environment. Another method for inferring HGT is to analyse codon usage bias, as the rumen protozoa show a significant skew toward A-T rich amino acids. First observed by Eschenlauer et al. (1998) a later study by Devillard et al. (1999) found that many enzymes isolated from rumen ciliates showed significant codon usage bias e.g., AAG was used to code for lysine in 82% and 67% of bacterial and fungal sequences, respectively, whereas in the protozoal xylanase AAA was used almost exclusively.

Further use of ‘omic technologies will allow for a more, in-depth and robust understanding of the rumen protozoa as such techniques reduce the difficulties associated with defining function in a mixed microbial ecosystem. Using next-generation sequencing (NGS) and a meta ‘omic approach, this study aimed to investigate the enzymatic activity of the rumen protozoa in terms of carbohydrate, protein and lipid metabolism. The resulting information will significantly enhance and clarify our understanding of rumen protozoal function, whilst providing much-needed sequence data for improvement of gene annotations for these largely understudied eukaryotes. Indeed, the generation of sequence data will enable better coverage of the protozoa in meta ‘omic studies of the rumen, reducing the risk of them being mis- and underrepresented, leading to more complete and accurate results.

## Materials and Methods

All experiments were conducted with the authority of Licenses under the United Kingdom Animal Scientific Procedures Act, 1986. The experiment was also approved by the Local Aberystwyth University Ethics Committee and reviewed and approved by the Animal Welfare and Ethical Review Body (AWERB) in line with University procedure.

Two liters of hand-squeezed and strained rumen fluid was obtained from three non-lactating, cannulated Holstein-Friesian cows. Cows were allowed 24 h access to grazing and free access to water. Samples were collected after 2 h after feeding (withdrawal from pasture and a small (250–500 g) concentrate incentive). Samples were pooled and transferred to an incubator at 39°C until use (samples were used within 1 h of collection; Huws et al., 2009).

Rumen fluid was then aliquoted into two 1 L burettes and the protozoa separated by gravity-fractionation as described by Huws et al. (2009). Per burette, 0.5 g of glucose was added and the apparatus incubated at 39°C for 1 h, during this time, the protozoa and many other microorganisms moved to the bottom of the burette in pursuit of the glucose where they were siphoned off into sterile 50 mL tubes. Samples were centrifuged at 100 × *g* for 10 min to remove the remaining plant material, followed by washing in Coleman’s buffer as described by Martin et al. (1994). A small portion of the pellet was diluted 1:10 using ddH_2_O and fixed for 2 h at room temperature with an equal volume of MFS solution [3.5% (v/v) formaldehyde and 8 g/L NaCl] before mounting 10 μL onto a slide and examining under a light microscope (Tymensen et al., 2012). The remainder was used for RNA extraction and downstream applications as outlined below (stored at −80°C).

### RNA Extraction

The remaining protozoal pellet was thawed and RNA extracted using the FastRNA Pro-Soil Direct kit^TM^ (MP Biomedicals, United Kingdom) according to the manufacturer’s guidelines. RNA concentration was quantified using the Epoch^TM^ Micro-Volume Spectrophotometer (Biotek, United States) before use of the Poly(A) Purist^TM^ MAG kit (Life Technologies, United Kingdom) to enrich polyadenylated mRNA, following the manufacturer’s guidelines. To remove any contaminating DNA prior to cDNA synthesis, a TURBODNA-free kit (Thermo-Fisher Scientific, United States) was used, following the manufacturer’s guidelines. To check for DNA contamination, a bacterial 16S rDNA (5′-AGAGTTTGATCCTGGCTCAG-3′ and 5′-ACGGGCGGTGTGTACAAG-3′) and eukaryotic 18S rDNA (5′-AGCCTGCGGCTTAATTTGAC- 3′ and 5′-CAACTAAGAACGGCCATGCA-3′) polymerase chain reaction (PCR) was carried out. PCR products were analyzed on a 1% agarose gel, with a 1 kB ladder and the gel was visually assessed for the presence of banding using the Gel doc system (Bio-rad, United Kingdom). Absence of bands was observed, indicating that the RNA was low in bacterial and fungal DNA contamination.

### Library Construction

The SMART^®^ cDNA library construction kit (Clontech Laboratories. Inc., United States) was used to create the metatranscriptomic library using LDPCR and the Advantage 2 PCR kit (Clontech Laboratories Inc., United States) following the manufacturer’s guidelines. The cDNA was digested using the *Sfil* restriction enzyme and size-fractionated using CHROMA SPIN-400 gel filtration columns (Clontech Laboratories. Inc., United States). Fractions containing the largest size and concentration of cDNA were pooled then cleaned via ethanol precipitation. The *Sfil* digested cDNA was then ligated into the λTriplEx2 vector (Clontech Laboratories Inc., United States) according to the manufacturer’s instructions.

For the primary titer, Luria Bertani (LB) broth with 10 mM MgSO_4_ and maltose (0.2%) was added to a liquid culture of *Escherichia coli* XL1-Blue and incubated at 37°C with 200 rpm shaking overnight to achieve an OD_600_ of 2.0. The culture was centrifuged at 5,000 × *g* for 5 min before resuspension in 7.5 mL of 10 mM MgSO_4_. The vector containing the cDNA was packaged immediately into the λ phage using the MaxPlax^TM^ Lambda Packaging Extract according to the manufacturer’s guidelines. To visualize the number of recombinant clones versus non-recombinants, blue-white color screening with 80 μg/mL Xgal (5-Bromo-4-chloro-3-indolyl-β-D-galactopyranoside) and 20 mM IPTG (Isopropyl β-D-1thiogalactopyranoside) was used. LB top agar (0.7% agarose) was combined with 300 μL of 1M MgSO_4_, X-gal and IPTG. The packaged reactions were diluted 1:10 and 2.5 μL of each was combined with 200 μL of *E.coli* XL1-Blue cells in 10 mM MgSO_4_ and incubated at 37°C for 15 min. This was added to 4 mL of LB top agar and overlaid onto LB/MgSO_4_ (10 mM) plates and incubated at 37°C overnight. Plates were visually assessed for blue and white plaques and counted to give a ratio of recombinants to non-recombinants and a pfu/mL.

A culture of XL1-Blue MRF’ *E. coli* was grown and resuspended in 10 mM MgSO_4_ as previously described, then adjusted to OD_600_ 0.5 using sterile 10 mM MgSO_4_. This was combined (500 μL) with the packaged reaction and incubated at 37°C for 15 min. Cooled LB/MgSO_4_ (10 mM) top agar was added, mixed and plated onto LB/MgSO_4_ agar plates. The plates were incubated at 37°C overnight. 1X Lambda dilution buffer (12 mL) was added to each plate followed by storage at 4°C overnight. The plates were then rocked at room temperature for 1 hour (h) before the bacteriophage suspension was recovered and pooled.

### Functional Screening

The first screen applied to the library was a functional assay for cellulolytic activity using carboxymethyl cellulose (CMC) as a substrate and Congo red for post-staining. *E. coli* XL1 Blue MRF’ cells were prepared and coupled with 2,000 pfu (5 μL) of the amplified library and incubated at 37°C for 20 min. LB top agar (8 mL) with MgSO_4_ and IPTG was added and mixed then spread onto LB agar with MgSO_4_. Cellulase (10 μL) from *Aspergillus niger* (Sigma-Aldrich, United States) (8 units/mL) was used as a positive control.

A layer of 0.2% (w/v) CMC, 0.7% (w/v) agarose and 25 mM Potassium phosphate buffer (pH 6.5) was overlaid onto each plate before incubation overnight at 37°C. Plates were then flooded with Congo red dye (1 mg/mL) for 30 min and rocked at 130 rpm. The Congo red dye was removed and 1 M NaCl solution added and rocked at 130 rpm for 20 min. The solution was discarded and the process repeated. Plaques expressing cellulolytic activity would appear yellow on the red background.

The second screen applied to the library was a functional assay for lipolytic activity using Tributyrin as a substrate and Spirit Blue as an indicator dye. Phage and bacterial cells were prepared at described above then spread on Spirit blue plates (Sigma-Aldrich, United Kingdom) supplemented with 1% Tributyrin. Phospholipase A_1_ from *Thermomyces lanuginosus* (Sigma-Aldrich) (10 KLU/g) was used as a positive control. The plates were incubated for 24 – 48 h at 37°C. Plaques expressing lipolytic activity would show a clear zone around their circumference.

The third screen applied to the library was a functional assay for protease activity using egg yolk agar, as described by Fu et al. (1997). The phage and bacterial cells were plated onto egg yolk agar and 2 μL of the protease from *Bacillus licheniformis* (Sigma-Aldrich, United States) (2.4 U/g) was used as a positive control. The plates were incubated for 2 – 4 days at 37°C, after which they were examined for plaques expressing proteolytic activity. A positive result would be indicated by off-white precipitation around the plaque.

### Sequencing

The protozoal cDNA library was prepared for sequencing using the Nextera^®^ DNA Library Preparation kit (Illumina, United States) according to the manufacturer’s instructions. Libraries were pooled and sequenced at 2 × 151 bp using an Illumina HiSeq 2500 rapid run, with samples duplicated over two lanes; procedures followed manufacturer’s instructions. Sequences resulting from this work can be found in NCBI’s Biosample database under accession number SAMN13506237.

**FIGURE 1:**
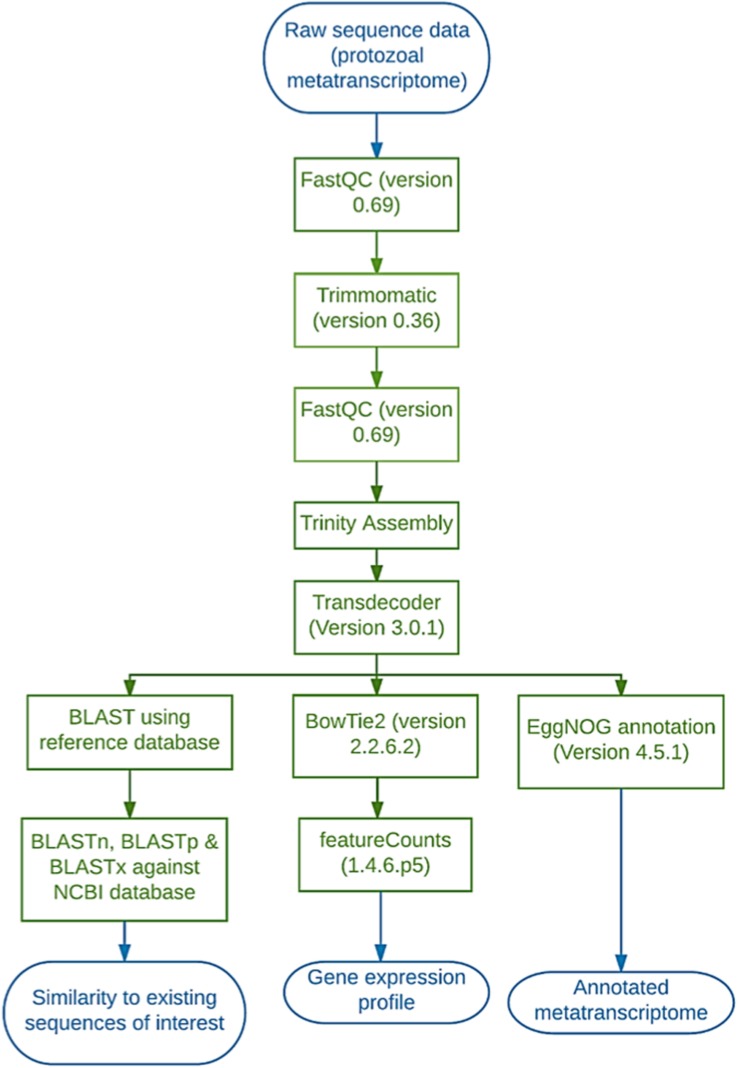
Diagrammatic representation of the workflow employed on the Galaxy platform to analyse the protozoal metatranscriptome.

Sequencing data were handled using the Galaxy platform (Version 17.01). Sequences were first aligned to the *Bos taurus* (ARS-UCD1.2) and *Lolium perenne* (ASM173568v1) genome using BowTie2 and any matches were removed from the dataset. The sequences were quality checked using FastQC (Version 0.69; Babraham Bioinformatics) (Andrews, 2010) then trimmed using a sliding window on Trimmomatic (Version 0.36.0) (Bolger et al., 2014) (Figure 1). The sliding window size was set at 4 bps and average quality required was set at 20 (given as a phred33 value this translates to 1 in 100 probability of an incorrect base call i.e., 99% accuracy. Trinity (Version 0.0.1) was used to assemble the sequences (using the paired-end option and default parameters) and then Transdecoder (Version 3.0.1 on default parameters) to identify coding regions. Transdecoder was run using the following parameters: Minimum protein length 100 bps, universal genetic code, retain long ORFs equal to or longer than 900 bps (300 aa) and train with the number of top longest ORFs: 500. EggNOG mapper (Version 4.5.1) was then used to annotate the sequences and Bowtie2 (Version 2.2.6.2) for alignment back to the Trinity assembly (paired library option with a built-in genome index, simple analysis mode and default parameters) (Figure 1). FeatureCounts (Version 1.4.6.p5) was used on default parameters to count the number of reads aligned to each functional category (Figure 1). The FeatureCounts output was then merged with the EggNOG annotation giving the number of reads per functional category (Figure 1). Carbohydrate-active enzymes were manually selected using the appropriate functional category (Carbohydrate Transport & Metabolism, G) and the sequence inputted into BLASTp (NCBI), to gain more information with regards to active domains and enzyme family/subfamily. In doing so, multiple results were produced for the same sequence and all sequences returned matches. As such, the match with the highest% identity and bit score were selected. Any match that fell below the following criteria was discarded: length (100 bp), Bit score (80) and percentage of identical matches (> 0%). The same process was used to investigate the few proteases and lipases that were identified. Carbohydrate-active enzymes that ranked highly (within the top 100 most expressed genes) were also inputted into the dbCAN2 meta server which uses HMMER, DIAMOND and Hotpep to provide more in-depth information on any carbohydrate-binding domains present in the query sequence (Zhang et al., 2018).

To analyse codon usage the CAIcal online tool was used (NCBI). The.cds output file from transdecoder was inputted in FASTA format and the “Mold, protozoan, Coelenterate. Mitochondrial and Mycoplasma/Spiroplasma” genetic code and Codon Usage output was selected alongside default parameters.

The nucleotide sequences of cellulases (annotated as ‘cellulase’ or ‘GH5’) and xylanases (annotated as endo-1,4-beta-xylanases, GH10 or GH11) from the sequence data were aligned using MEGA7 (v. 7.0.26; Kumar et al., 2016) with enzymes from the same GH family produced by rumen protozoa, fungi and bacteria (sequences retrieved from GenBank). Phylogenetic trees were constructed using neighbor-joining clustering method (Saitou and Nei, 1987). A bootstrap consensus tree was inferred from 500 replicates, and branches corresponding to partitions reproduced in less than 50% bootstrap replicates are collapsed. The evolutionary distances were computed using the maximum composite likelihood method and are in the units of the number of base substitutions per site (Tamura et al., 2004). The analysis for xylanase genes involved 30 nucleotide sequences and there was a total of 241 positions in the final dataset. The analysis for cellulase genes involved 32 nucleotide sequences and there was a total of 70 positions in the final dataset. All positions containing gaps and missing data were eliminated.

## Results

### Protozoal Diversity

Microscopic examination of the protozoal pellet used to extract RNA suggested a mixture of type A and type B protozoa (Eadie, 1962; Dehority, 1993) (Supplementary Figures S1, S2). As samples from three different animals were pooled together, animals may have possessed different types. The majority of protozoa in the samples were entodiniomorphids: *Ostracodinium* (species likely to be *dilobum*) (Supplementary Figures S1A,B), *Diploplastron* (species *affine*) (Supplementary Figure S1C) *Entodinium* (species *furca monolobum*) (Supplementary Figure S1D), *Eudiplodinium* (species *maggii*) (Supplementary Figures S1E,F) and *Epidinium* (species *caudatum* and *quadricaudatum*) (Supplementary Figures S1G,H) (Dehority, 1993; Williams and Coleman, 2012). The holotrichous representatives observed were identified as *Isotricha* (species *intestinalis*) and *Dasytricha* (species *ruminantium*) (Supplementary Figures S2J,I) (Dehority, 1993; Williams and Coleman, 2012).

### RNA Purity

Protozoal RNA was confirmed as sufficiently pure and free from contaminating DNA as evidenced by the absence of PCR products for both the 16S rDNA bacterial and eukaryotic 18S rDNA PCR (Supplementary Figure S3).

### Functional Phage-Based Assays

**FIGURE 2:**
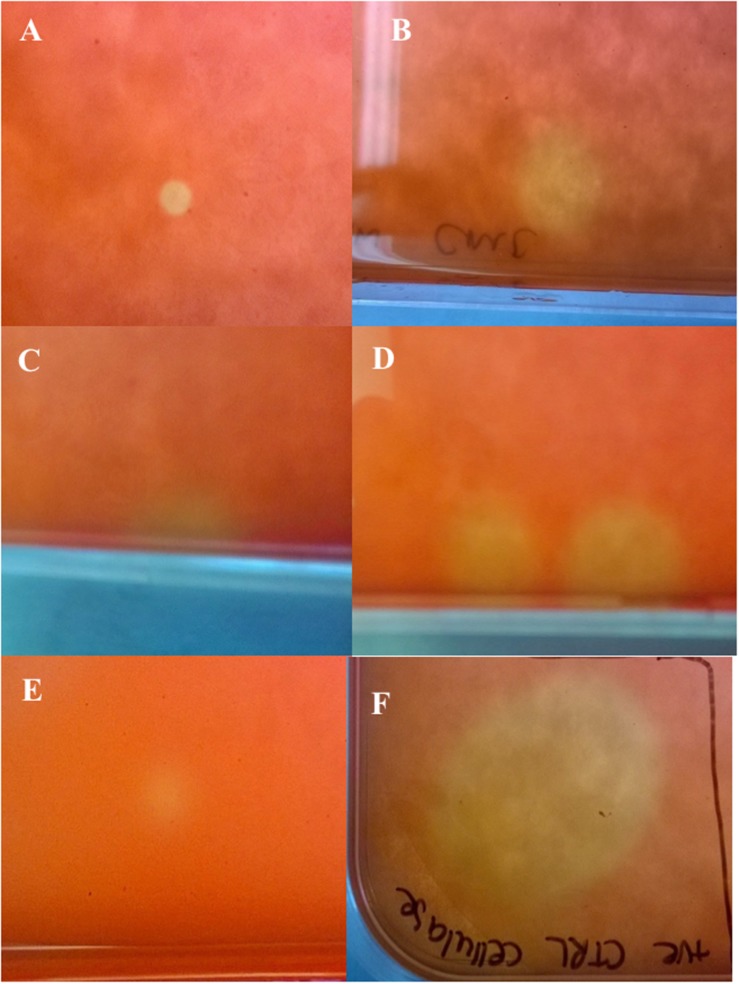
Amplified cDNA library plated on *Escherichia coli* lawn with carboxymethyl cellulose overlay and post-staining with Congo red (Primary assay). **(A–E)** Cellulase positive plaques indicated by yellow coloring; **(F)** Positive control (cellulase from *Aspergillus niger*) indicated by yellow coloring.

The library produced a titer of 6.5 × 10^6^ pfu/mL and recombinant plaques accounted for 15–20% of total plaques. Primary screening of the amplified cDNA library using substrates for cellulase, lipase and protease activities, yielded mixed results. The only putatively positive results were observed in response to CMC substrate, which consistently revealed six positive plaques in addition to the positive control post-staining indicating cellulase activity (Figure 2). No positive results were detected using differential media to screen for lipases and proteases. Viral plaques were observed but showed no zones of clearance or colorimetric change in any instances. Positive controls indicated that the assays were functional.

### Sequencing and Bioinformatics

Direct sequencing of the metatranscriptome of the protozoal cDNA resulted in approx. 3.3 million raw reads and 3.1 million trimmed reads (Table 1). The metatranscriptome sequences were reconstructed using Trinity on Galaxy, which provided 9,101 contigs (Table 1). After applying TransDecoder 2,505 genes were predicted, of these EggNOG annotated 968 (Table 1).

**TABLE 1:** Protozoal metatranscriptome sequencing data.

Data	No. sequences
Raw data	3,277,915
Trimmed data	3,125,731
Contigs assembled	9,101
Predicted genes	2,505
Annotated genes	968
No. Reads aligning to contigs	402,226

**FIGURE 3:**
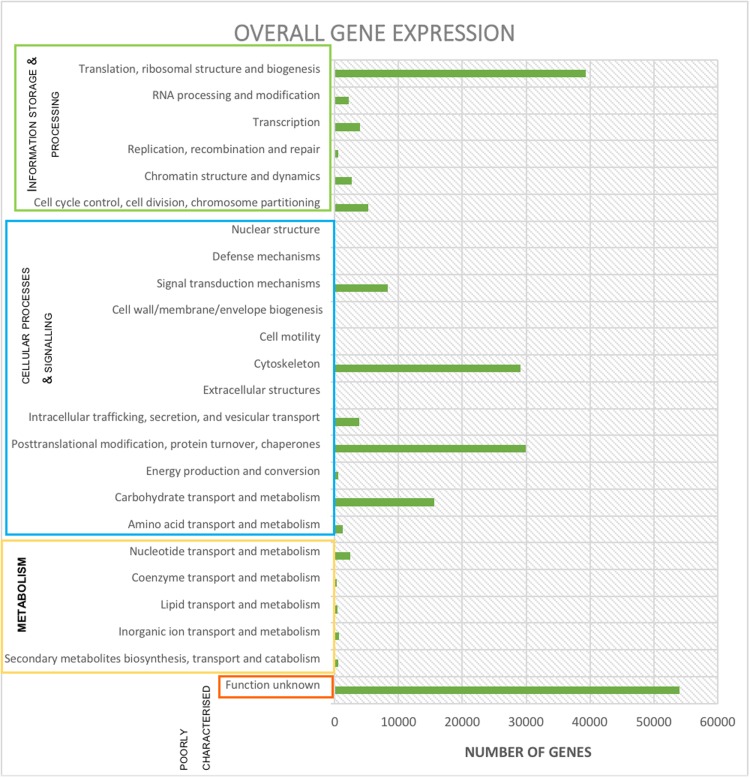
Expression (reads) per functional subcategory from the protozoal metatranscriptome using Bowtie2, FeatureCounts and EggNOG online mapper. Subcategories are collated into the appropriate main category.

Use of BowTie2 resulted in 402,226 reads aligned to contigs, which when processed by FeatureCounts resulted in 201,113 reads aligning to predicted genes (Table 1). When summarized in terms of hierarchical subsystem functional categories the most highly expressed were: ‘translation, ribosomal structure & biogenesis,’ ‘post-translational modification, protein turnover & chaperones,’ ‘the cytoskeleton’ and ‘carbohydrate transport & metabolism’ (Figure 3). Although, by far the category containing the largest number of expressed reads was ‘poorly characterized’ in which function was unknown (Figure 3).

**FIGURE 4:**
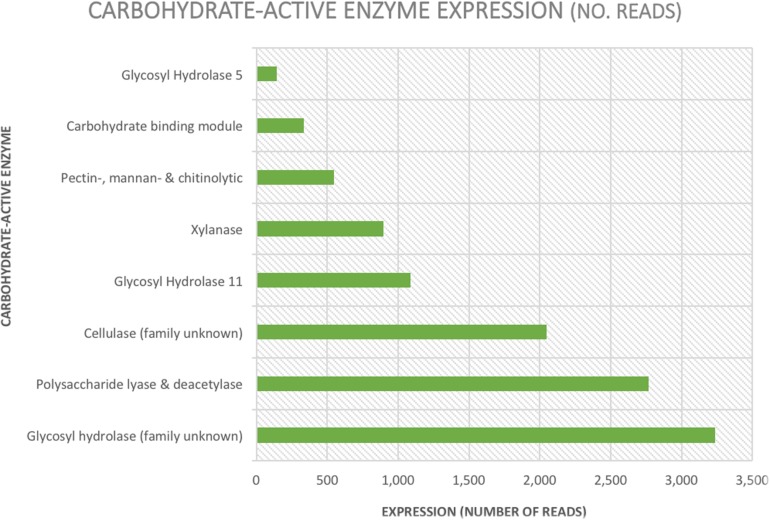
Graphical representation of expression levels (number of reads) of carbohydrate-active enzymes annotated by EggNOG and confirmed using NCBI’s online Basic Local Alignment Search Tool.

The total number of carbohydrate-active enzymes was 16,248 (8% of annotated reads) with a large proportion (3,239) being glycosyl hydrolases from an unknown family (20% of all reads involved in carbohydrate metabolism and transport) (Table 2 and Figure 4). Target sequences annotated as carbohydrate-active by EggNOG (in both the functional category and gene description) were further characterized using BLASTp (Table 2 and Figure 4). After selecting results according to criteria laid out in section 2.4, 9 sequences matched a glycoside hydrolase (Family 5) from an uncultured equine cecal ciliate (Sequence ID: CDG46723), 3 sequences matched a xylanase produced by *E. maggii* (Sequence ID: CAL91982), 2 matched a xylanase produced by *P. multivesiculatum* (Sequence ID: CAD56867) and 2 matched to an endo- 1, 4- beta-xylanase also produced by *P. multivesiculatum* (Sequence ID: BAA76395). Other matches were made to various endo-1, 4-beta-xylanases, xylanases, cellulases and endoglucanases from rumen microorganisms (three matches to rumen bacteria and 5 to rumen protozoa; sequence IDs: SCI73846.1; WP_027622710.1; GAE88377; BAA76394; BAA76395; CAL91973; CAL91979; CAH69214).

**TABLE 2:** Expression (number of reads) and percentage of total reads per type and family of carbohydrate active enzyme.

Enzyme	Expression (reads)	% of total reads
All carbohydrate-active enzymes	16,248	8
Glycosyl hydrolase (family unknown)	3,239	1.6
Polysaccharide lyase & deacetylase	2,767	1.4
Cellulase (family unknown)	2,046	1
Glycosyl Hydrolase 11	1,090	0.5
Xylanase	898	0.45
Pectin-, mannan- & chitinolytic	546	0.3
Carbohydrate-binding module	337	0.17
Glycosyl Hydrolase 5	146	0.07

Using the sequences as queries for a BLASTp alignment against dbCAN helped to give a more comprehensive overview of the glycosyl hydrolases found in this dataset. Only four enzymes were commonly identified by all three tools used by dbCAN, only two of which fell within the top 100 most expressed genes in the dataset and were of some interest. Two of the three tools were able to commonly identify 27 enzymes – 1 by Diamond + HMMER and 26 by Hotpep + HMMER. The two highest-ranking carbohydrate-active enzymes in the dataset (14^th^ and 43^rd^) were designated CBM79 by HotPep and HMMER and were of most interest here. This protein is produced by *Ruminococcus* sp. and falls into the GH 9 family, which binds various β-glucans. Other carbohydrate-active enzymes within the top 100 most expressed enzymes were from glycosyl hydrolase (GH) family 11 (xylanases) and 45 (endo-glucanases).

Using BLASTp and dbCAN, putative proteolytic enzymes identified were categorized as Cathepsin B and a cysteine protease (Cathepsin F). Cathepsin B ranked as the 37^th^ most expressed enzyme and showed 67% identity with a hypothetic protein produced by the protozoan parasite *Ichthyophthirius multifiliis* (Sequence ID: XM_004027641). Cathepsin F was ranked at 41 in terms of expression and showed 43% homology with a hypothetical protein from *Halomonas* sp. (Sequence ID: WP_110285651). Two lipases of the GDSL family were identified by BLASTp but were not highly expressed. One showed 53% homology with a lipase from the GDSL family produced by *Prevotella ruminicola* (accession number: EGD48497.1) and the other 51% similarity with a GDSL-family lipase from *Clostridium papyrosolvens* (accession number: ADE81025.1).

**FIGURE 5:**
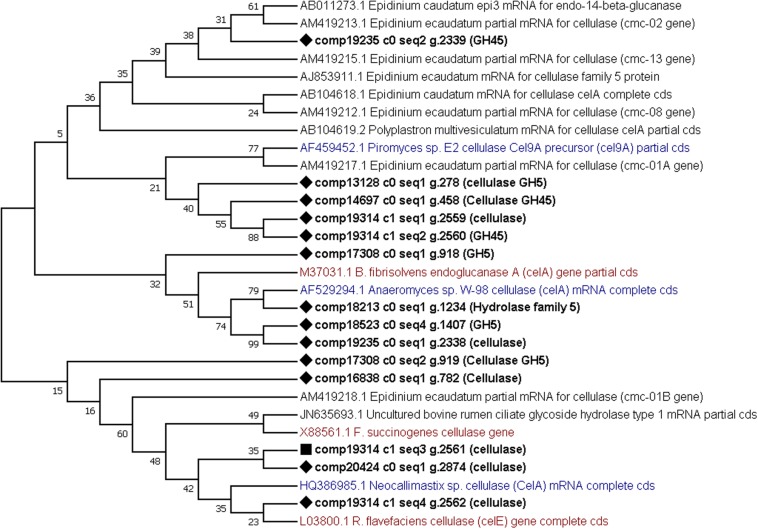
Comparative phylogenetic tree of all putative cellulase (or GH5) genes detected within the protozoal metatranscriptome. Twelve representative protozoal sequences from *Epidinium ecaudatum* (6), *Epidinium caudatum* (4), *Polyplastron multivesiculatum*, (1) and an uncultured bovine rumen ciliate (1), three fungal cellulases produced by *Neocallimastix* sp., *Piromyces* sp. and *Anaeromyces* sp. and three bacterial cellulases from *Ruminococcus flavefaciens, Butyrivibrio fibrisolvens* and *Fibrobacter succinogenes* are given for comparison. Target sequences from the metatranscriptome are shown in bold with a diamond shape node marker, the most highly expressed enzyme is marked with a square, protozoal sequences are given in black, bacterial in red and fungal in blue text.

**FIGURE 6:**
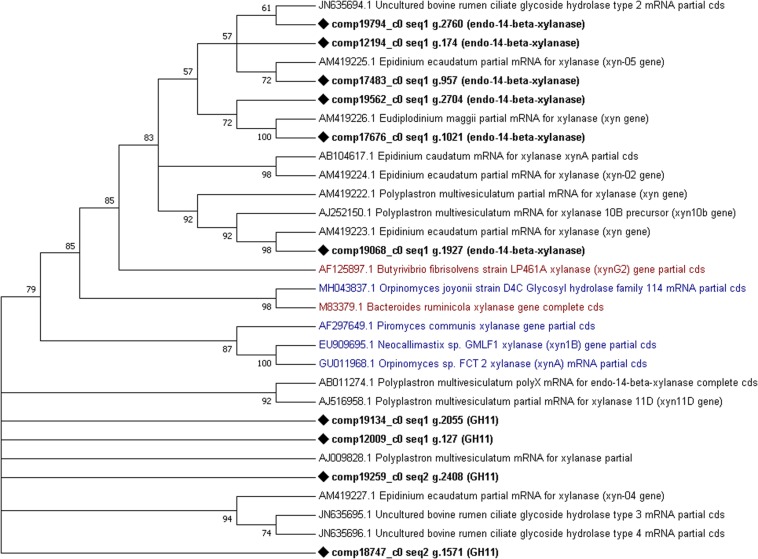
Comparative phylogenetic tree of all putative xylanase (GH10 or GH11) genes detected within the protozoal metatranscriptome. Fourteen representative protozoal sequences from *Epidinium ecaudatum* (4), *Polyplastron multivesiculatum* (5), uncultured bovine rumen ciliates (3) *Epidinium caudatum* (1) and *Eudiplodinium maggii* (1), three fungal cellulases produced by *Neocallimastix* sp., *Piromyces* sp., *Orpinomyces* sp. and *Orpinomyces joyonii* and two bacterial cellulases from *Bacteroides ruminicola* and *Butyrivibrio fibrisolvens* are given for comparison. Target sequences from the metatranscriptome are shown in bold with a diamond shape node marker, the most highly expressed enzyme is marked with a square, protozoal sequences are given.

Finally, phylogenetic trees constructed from cellulases and xylanases identified from this dataset (target sequences) and sequences retrieved from GenBank (GH5, cellulase, xylanase, GH10 and GH11 sequences from the rumen protozoa, bacteria and fungi) to visualize their similarity. From the phylogenetic tree constructed using cellulase sequences it can be observed that several (7) target sequences cluster together with some similarity shown to AF459452 *Piromyces* sp. Cel9A (Figure 5). One target sequence shows some similarity to other fungal enzymes produced by *Anaeromyces* sp. (AF529294) and *Neocallimastix* sp. (HQ386985) (Figure 5). The remaining 6 target sequences showed similarity to those produced by *E. ecaudatum* (AJ853911 and AM419212), *E. caudatum* (AB104617) and uncultured bovine rumen ciliates (JN635693) (Figure 5). The phylogenetic tree constructed from xylanase sequences shows clustering of fungal genes (GU011968, EU909695, and AF297649) and of a fungal and bacterial gene (M83379 and MH043837) (Murphy et al., 2019) (Figure 6). Four target xylanase genes show some, limited similarity to xynD11 and xylanases produced by *P. multivesiculatum* (AB011274, AJ516958, and AJ009828) (Figure 6). Five target xylanases showed close similarity to those produced by *E. ecaudatum*, *E. maggii* and an uncultured bovine rumen ciliate (AM419223, AM419225, AM419226, and JN635694) (Figure 6).

### Codon Usage Bias Analysis

Analysis of codon usage in the data set reveals a clear bias toward AAA, AAT, GAT and GAA all of which are used in >5% of the metatranscriptome (Table 3). Codons AAA and GAA were used in 12.61 and 11.5%, respectively, and together account for 27% of all amino acids in this dataset. There is also a bias toward several other A-T rich codons, all of which are used 2 – 5% of the time (i.e., TAT, TTA, TTT, GTT, CAA, ACT, AGA, ATA, and ATT) (Table 3). A and T constituted 69% of the genome and G and C accounted for 31%. This is a lower GC content than detected by the FastQC program due to trimming (43% pre-trimming, 31% post-trimming).

**TABLE 3:** Codon usage for sequence data obtained for the cDNA library.

CODON	AAA	AAC	AAG	AAT	ACA	ACC	ACG	ACT	AGA	AGC	AGG	AGT	ATA	ATC	ATT	CAA
**AMINO ACID**	**K**	**N**	**K**	**N**	**T**	**T**	**T**	**T**	**R**	**S**	**R**	**S**	**I**	**I**	**I**	**Q**

Total	44568	2713	7012	17589	5804	1757	300	9210	12379	1220	977	4926	7723	1968	11518	11781
%	12.61	0.77	1.98	4.99	1.64	0.50	0.08	2.61	3.50	0.35	0.28	1.39	2.19	0.56	3.26	3.33

**CODON**	**CAC**	**CAG**	**CAT**	**CCA**	**CCC**	**CCG**	**CCT**	**CGA**	**CGC**	**CGG**	**CGT**	**CTA**	**CTC**	**CTG**	**CTT**	**GAA**

**AMINO ACID**	**H**	**Q**	**H**	**P**	**P**	**P**	**P**	**R**	**R**	**R**	**R**	**L**	**L**	**L**	**L**	**E**

Total	638	754	3854	8153	1160	377	5464	258	144	123	837	1127	895	271	4144	40636
%	0.18	0.21	1.09	2.31	0.33	0.11	1.55	0.07	0.04	0.03	0.24	0.32	0.25	0.08	1.17	11.50

**CODON**	**GAC**	**GAG**	**GAT**	**GCA**	**GCC**	**GCG**	**GCT**	**GGA**	**GGC**	**GGG**	**GGT**	**GTA**	**GTC**	**GTG**	**GTT**	**TAC**

**AMINO ACID**	**D**	**E**	**D**	**A**	**A**	**A**	**A**	**G**	**G**	**G**	**G**	**V**	**V**	**V**	**V**	**Y**

Total	2657	4158	19199	5263	2405	291	8271	13534	936	915	7109	5618	1892	777	8949	1259
%	0.75	1.18	5.43	1.49	0.68	0.08	2.34	3.83	0.26	0.26	2.01	1.59	0.54	0.22	2.53	0.36

**CODON**	**TAT**	**TCA**	**TCC**	**TCG**	**TCT**	**TGA**	**TGC**	**TGG**	**TGT**	**TTA**	**TTC**	**TTG**	**TTT**	**ATG**		

**AMINO ACID**	**Y**	**S**	**S**	**S**	**S**	**W**	**C**	**W**	**C**	**L**	**F**	**L**	**F**	**M**		

Total	9584	5502	1076	319	4750	36	1783	2465	2795	12480	4097	1704	7085	6260		
% Usage	2.71	1.56	0.30	0.09	1.34	0.01	0.50	0.70	0.79	3.53	1.16	0.48	2.00	1.77		

*Data shows the number of times a codon occurred in the data and converted to % occurrence.*

## Discussion

With the view to developing the current understanding of the role and functions of the rumen protozoa, this study created a phage-based metatranscriptomic library from purified protozoal cDNA that was functionally screened for lipase, protease and cellulase activity. Six positive plaques were identified in response to CMC agarose, but no activity against tributyrin/spirit blue and egg yolk agar was observed (indicative of lipolytic and proteolytic activity, respectively). Direct sequencing data confirmed the functional screening results by demonstrating that carbohydrate metabolism is a primary function of the rumen protozoa, especially when compared with lipolytic and/or proteolytic activity. The sequencing data also revealed high expression levels of chitinases, which are likely utilized by rumen protozoa to digest rumen fungi which possess chitin-rich cell walls (Morgavi et al., 1994). This study suggests that the primary roles of the rumen ciliates are predation and carbohydrate metabolism.

The majority of studies concerning the carbohydrate-active enzymes of the rumen protozoa span the period between 1960 and 2000, pre-dating the genomic era (Guttmacher and Collins, 2003; Konstantinidis et al., 2006). As such, very little sequence data are available and most research uses faunated vs. defaunated animals or simple agar-based assays with pure cultures to infer activity. The only other published metagenomic library to be constructed exclusively from rumen protozoa was that by Findley et al. (2011) using similar methods to those described here. Major differences between the studies include the kits and protocols used to construct the metagenomic library and the use of *in vivo* excision, which was not possible in the current study. Excising the phagemid vector using *in vivo* excision allows for transformation into eukaryotic cells which would allow functional screening followed by PCR and Sanger sequencing. The use of phage in functional assays is more complex when compared to other vector systems and is not widely used, as any one plaque may contain up to 1 million individual phages, of which only one or two may be expressing the desired activity. On the other hand, using a bacterial or eukaryotic vector allows for more straightforward functional screening in terms of both working conditions and identification of positive colonies. Other studies have utilized cDNA libraries to characterize single species of protozoa, which have produced mostly partial and one complete gene encoding carbohydrate-active enzymes (Wereszka et al., 2004, 2006; Bełżecki et al., 2007). More recently, NGS has allowed some partial mRNA to be deposited in Genbank for GH 5 and 9 as well as uncategorized cellulases and xylanases produced by ruminant protozoa, but no characterization or further work has been done (e.g., BAC57894; BAC57895; CAL91983; CAL91968). The 2019 study by Wang et al. (2019) provides the first transcriptome of a single rumen ciliate, *Entodinium caudatum*, using similar methods to those detailed here. The study uncovered numerous carbohydrate-active enzymes (including lysozymes and chitinases) in agreement with the present experiment Such studies have provided a basis for the work described here, which utilizes ‘omic technologies to elucidate the role and function of the rumen protozoa, provide more in-depth information about the enzymes they produce and to investigate the potential for HGT.

The use of NGS provides many advantages over traditional culture-based methods, the primary benefit being the bypassing of establishing and maintaining a culture of protozoa. Rumen protozoa are notoriously difficult to maintain in culture and although decades of optimization have resulted in improved methods, it remains impossible to maintain them in axenic culture (Newbold et al., 2015). The use of direct sequencing also negates issues with contamination; the rumen protozoa are well-known for engulfing bacterial and fungal cells which can significantly skew assays, rendering results inaccurate and unreliable (Belanche et al., 2012; Williams and Coleman, 2012; Newbold et al., 2015). The use of NGS allows protozoal RNA to be extracted and thoroughly purified (here, Poly(A) enriched, DNase treated and Lithium chloride precipitation was used) as well as assessed for the presence of contaminating DNA using PCR and gel electrophoresis before sequencing. After sequencing, there is also the option to align data to various genomes (host, plant, fungal, bacterial etc.) and bin any matching results which give complete certainty that the sequences retained are purely protozoal. Utilizing these techniques, this study approached the rumen protozoa from both a functional and sequence-based perspective to give a well-rounded picture of their role in the rumen. The main drawback of this approach in terms of the rumen protozoa is the lack of reference sequences available, in large meta’ omic datasets this can lead to mis- and underrepresentation, particularly when the concept of horizontal gene transfer is taken into consideration. To combat these limitations, the generation of sequence data and its subsequent publication is key, not only will this improve the accuracy of meta ‘omic datasets produced from the rumen but will further our understanding of the rumen protozoa and their role in the rumen.

Illumina sequencing revealed a wealth of carbohydrate-active enzymes in addition to those that specifically target the rumen fungi (and in some cases bacteria). Overall, 8% of all reads from the metatranscriptome were involved in carbohydrate metabolism or transport. However, 20% of carbohydrate-active enzymes identified were annotated as ‘GH, family unknown.’ Such a result serves to highlight the distinct lack of sequence data available for the rumen protozoa and calls into question whether this result may even be an underestimation in itself (Comtet-Marre et al., 2017). When approaching the metatranscriptome as a whole, this issue becomes more obvious as EggNOG was unable to annotate 27% of predicted genes. Such a result highlights the lack of data available for the rumen protozoa but also adds value to that presented here, which could contribute significantly to our current understanding and future sequence-based work.

A range of glycosyl hydrolases were identified (from families 5, 25, 16, 43, 45, and 97) as well as endo-1, 4-β -xylanases (belonging to the glycosyl hydrolase 11 family). Two of these enzymes: glycoside hydrolase family 11 and polysaccharide deacetylase are ranked in the top 20 most expressed genes in the whole metatranscriptome. Polysaccharide deacetylases accounted for 1.4% of the entire metatranscriptome and were the 18^th^ most expressed gene. Polysaccharide deacetylases fall into carbohydrate esterase family 4 and catalyze the hydrolysis of N- or O-linked acetyl groups from polysaccharide residues (Arnaouteli et al., 2015). These enzymes are active against plant polysaccharides but are also used by bacteria to modify peptidoglycan in their cell walls to adjust to varying environmental conditions (Kobayashi et al., 2012). When subject to BLAST, this protein showed similarity (98%) to a glycosyl hydrolase family protein from *R. flavefaciens*. Analysis using the dbCAN metaserver identified the protein as carbohydrate-binding module 79 (CBM79); found specifically in the *R. flavefaciens* cellulosome, this forms part of a GH 9 catalytic module with endo-glucanase activity. However, with an AT composition of 67% it is highly likely that this enzyme has been horizontally acquired from the rumen bacteria to act against plant material and potentially against rumen bacteria upon engulfment (Garcia-Vallvé et al., 2000; Ricard et al., 2006; Kelly et al., 2009).

Some of the most abundant enzymes were the cellulases, or GH5s. When aligned to known protozoal sequences, several (7) of the sequences grouped away, showing no similarity, instead the phylogenetic tree indicated a relationship with cel9A from *Piromyces* sp. (AF459452) and cellulases from *R. flavefaciens* and *B. fibrisolvens* (L03800 and M37031, respectively). This highlights novelty in the enzymes identified here and supports the hypothesis that many carbohydrate active enzymes utilized by the protozoa have been horizontally acquired.

Xylanases, mostly endo-1, 4-beta-xylanases (EC 3.2.1.8 of the GH11 family), were also highly expressed - this is in keeping with current literature where the protozoa have been shown to produce several, highly active xylanases (Orpin, 1984; Williams and Coleman, 1985; Devillard et al., 1999, 2003; Béra-Maillet et al., 2005). Many of the xylanases identified in this dataset matched those described by McEwan et al. (2008); Findley et al. (2011) and/or Béra-Maillet et al. (2005) which is demonstrated in the phylogenetic trees. Some xylanases (4) did not group with known sequences in the phylogenetic tree, suggesting novelty.

The addition of glucose during fractionation has the potential to skew results and affect the expression of monosaccharide degrading enzymes, however, comparatively few enzymes involved in glucose metabolism were identified. Examples include glucokinase (K00845), aldose 1-epimerase (KO1785) and glucose phosphoglucomutase (K01835), none of which were highly expressed ranking 551^st^, 863^rd^ and 302^nd^ in the dataset in terms of expression. Far more numerous were those responsible for the breakdown of polysaccharides.

The metatranscriptome also revealed chitin and pectin degrading enzymes, generally thought to be produced by the rumen bacteria and fungi. It has been demonstrated that many enzymes belonging to the rumen microbes have been acquired or shared via horizontal transfer (Garcia-Vallvé et al., 2000; Ricard et al., 2006; Kelly et al., 2009). This is highly likely in the case of the rumen protozoa, as they are prolific in their capture and digestion of rumen bacteria and fungi, placing these microbes in very physical contact which increases the chance of an exchange. Previous studies suggested that some protozoa may produce chitinases. Miltko et al. (2012) found two endo-chitinases and two exochitinases produced by *E. maggii* but the evidence is scarce in comparison to that for the rumen bacteria and fungi. Strains of *Clostridia* have been proven to possess a variety of chitinases, showing activity lyzing the fungal cell wall and inhibiting the fungal β- 1, 4- endoglucanases (Kopečný et al., 1996; Kopečný and Hodrová, 2000). This may be perceived as evidence of direct competition between the rumen fungi and bacteria and in the case of the protozoa as a means of digestion inside the food vacuole (Morgavi et al., 1994). Two chitinases were identified in this dataset, one of which showed similarity to a hypothetical protein produced by *Butyrivibrio* sp. (90%). Another showed 95% identity with the GH18 chitinase-like family of enzymes produced by the *Eubacterium* genus. This result is consistent with the current literature and contributes toward the hypothesis that the rumen protozoa may have acquired chitinolytic enzymes from neighboring bacteria.

On the other hand, the production of pectin-active enzymes can be attributed solely to the need to break down polysaccharides in plant material. This is a largely recalcitrant compound used by the plant to give physical strength and to act as a barrier; as such, this makes it difficult to degrade (Voragen et al., 2009). In a study by Gradel and Dehority (1972), seven rumen bacteria were found to degrade solubilized and purified pectin in pure culture and Dušková and Marounek (2001) found consistent activity against pectin in *P. ruminicola*. In addition to the rumen bacteria, fungal species *Orpinomyces* and *Neocallimastix* produce pectinolytic enzymes: polygalacturonase and pectin lyase (not pectinesterase) (Kopečný et al., 1996). In the present study, sequencing revealed two of the four classes of pectin-active enzymes: pectinesterases and pectate lyases. When subject to BLAST, pectate lyases showed similarity to those found in *Piromyces* sp. *R. flavefaciens* and *Neocallimastix californiae* whilst the pectinesterase identified showed some similarity to that produced by *Ruminococcus albus.*

Of the few proteases identified in this dataset, both ranked in the top 50 most expressed enzymes and were predicted to display lysosomal activity (particularly cathepsin B). The most likely use for this enzyme is in the breakdown of ingested bacteria. This supports the observation that the rumen protozoa predate bacteria and are well adapted to digest them (Newbold et al., 2015). Two lipases of the GDSL family were identified but were not highly expressed (one with just 43 reads and the other with just 22). This lack of both sequences and expression levels may suggest that the rumen protozoa do not directly contribute to ruminal lipid metabolism. Instead, this result supports that reported by Huws et al. (2009) whereby due to the ingestion of chloroplasts the rumen protozoa are rich in polyunsaturated fatty acids, potentially protecting them from biohydrogenation. In terms of protein metabolism, this data suggests that protozoal contribution stem mostly from the breakdown of their fellow microbes. However, it is important to note that the diet has a significant effect on the rumen microbiome (Kim et al., 2008; Huws et al., 2010; Petri et al., 2013; Ross et al., 2013). In this study, animals were allowed 24/7 access to pasture with no silage or concentrates, which may affect the results observed. In general, perennial ryegrass contains a good amount of protein (16-25%), especially when compared to silage, however, concentrates such as soya bean meal contain much higher levels (approx. 47%) (Germinal, 2015). Indeed, the vast majority of lipids in pasture are phospholipids (polyunsaturated fatty acids), whilst lipid supplementation in the TMR varies considerably depending on production values/aims (Elgersma et al., 2003; Rasmussen and Harrison, 2011). As such, there is strong potential for dietary effects to influence the activity of the rumen protozoa – to address the issues, future research may look to profile the protozoa under different dietary conditions and over different time points, for example.

Results from codon usage analysis showed significant usage bias in lysine and glutamic acid. Lysine was coded for by AAA in 12.61% of cases, compared to AAG which was used in just 1.98% of cases. Glutamic acid was coded for by GAA 11.5% of the time, compared to GAG which was used in 1.18% of cases. Bias can also be observed in coding for asparagine, where the AAT codon was used 4.99% of the time, whilst AAC was employed in just 0.77% of cases. Overall, 70% of the total sequences consisted of A and T. This codon bias is demonstrated throughout the metatranscriptome and also persists in genes that show similarity at the protein level to those produced by bacteria, maintaining a 60–70% A-T content in individual genes. This extreme nucleotide bias is observed in both coding and non-coding regions but the reason behind this sway toward AT-rich codons is yet to be elucidated. This skewed codons usage that still follows the “universal” codon usage pattern, are in keeping with previous work (McEwan et al., 2000b). In this previous work it was shown that unlike some ciliates such as *Tetrahymena*, the rumen ciliates do not use TAG and TAA to encode glutamic acid, but rather they use them together with TGA as stop codons. Furthermore, given the high percentage of A-T present in their genomes, TAA is the most abundant stop codon used by these organisms. In addition, beyond the coding region of messages from rumen ciliates, there is a general absence of AAUAAA the “universal” polyadenylation signal. Instead, the short 3’ untranslated region (typically < 100 nucleotides) contains a similar consensus sequence AUAAA (McEwan et al., 2000a).

This study has provided valuable insight into the metabolic contribution of the rumen protozoa. Their considerable contribution to carbohydrate breakdown has been observed, as well as some enzymes that were not thought to be commonly produced by rumen protozoa (pectinesterases, cathepsins and some glycosyl hydrolase families). Additional evidence of extensive HGT has been introduced, strengthening the argument that many genes have been acquired from the rumen bacteria and fungi that have enabled the protozoa to adapt to the carbohydrate-rich environment of the rumen. Evidence of adaptation to predation has also been presented, enzymes whose function are most likely in the digestion of rumen fungi and bacteria have been uncovered (pectin-active enzymes and cathepsins), cementing the role of the protozoa as predators in the rumen. The greatest limitation of this study is the use of a single time point, however, this information provides a sound basis for further research in terms of both the results presented and methods described. Nevertheless, this snapshot provides a wealth of new data which will further our understanding of the rumen protozoa, their functions and their role in the rumen environment in addition to providing valuable and much-needed reference sequences. This research may also serve as a guide or starting block for more in-depth research using the latest ‘omic techniques to improve our knowledge of the protozoa.

## Data Availability Statement

The datasets generated for this study can be found in the Biosample database under SAMN13506237.

## Ethics Statement

The animal study was reviewed and approved by the local Aberystwyth University Ethics Committee and the Animal Welfare and Ethical Review Body (AWERB) in line with University procedure. Experiments were conducted with the authority of Licenses under the United Kingdom Animal Scientific Procedures Act, 1986.

## Author Contributions

CW and SH conceived and designed the experiments. CW performed the experiments with support from PR, SH, and NM. CW were carried out the bioinformatic analyses with guidance from CC and BT. CW wrote the manuscript with revisions from SH. All authors have read and approved the final manuscript.

## Conflict of Interest

The authors declare that the research was conducted in the absence of any commercial or financial relationships that could be construed as a potential conflict of interest.
