# Supplementary figures and images for: Rumen Protozoa Play a Significant Role in Fungal Predation and Plant Carbohydrate Breakdown
Source: Front Microbiol. 2020 Apr 29;11:720. doi: 10.3389/fmicb.2020.00720 (PMC7200989; doi:10.3389/fmicb.2020.00720)

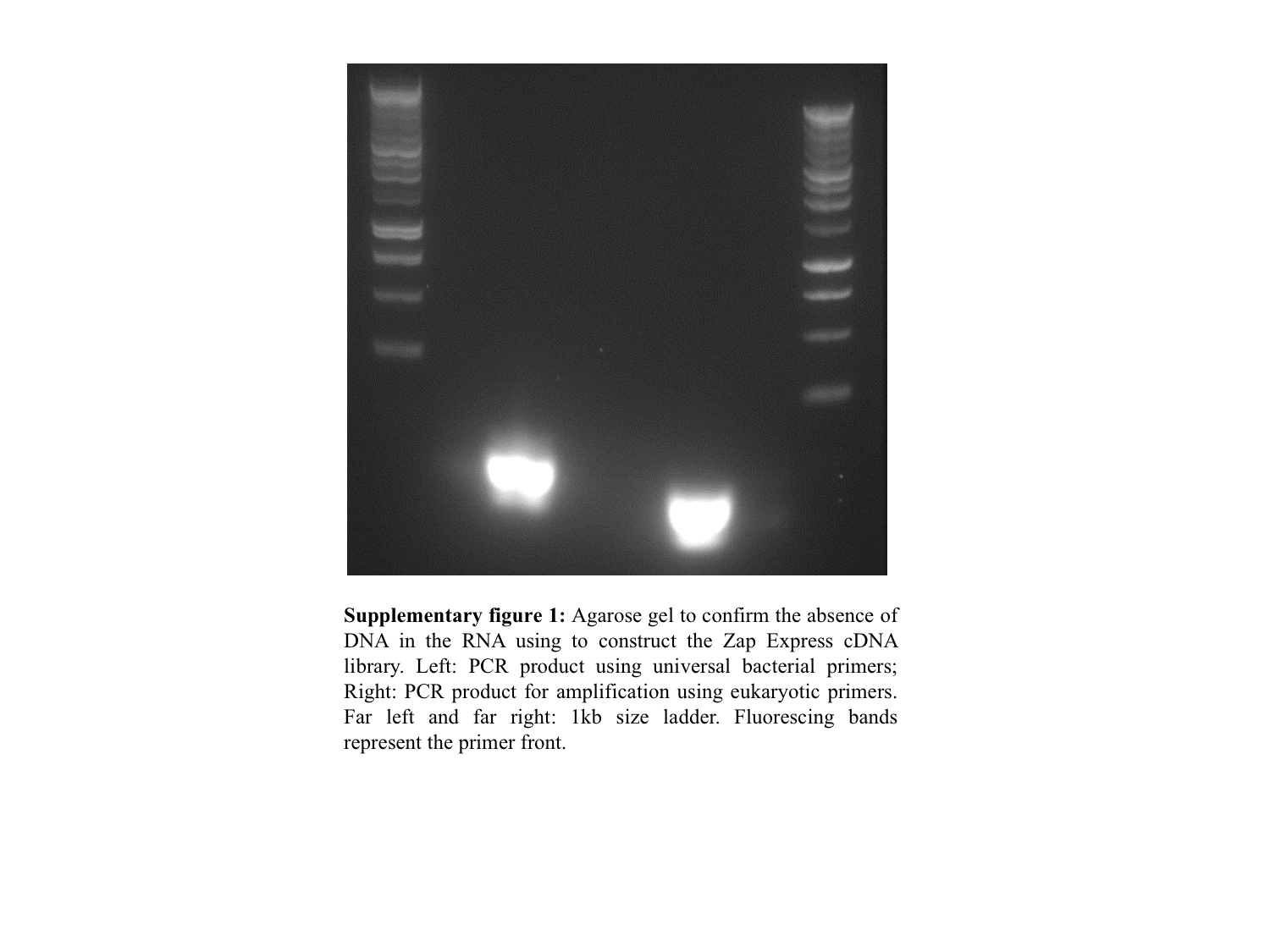

Supplement: Supplementary file 1 [file Image_1.tiff]

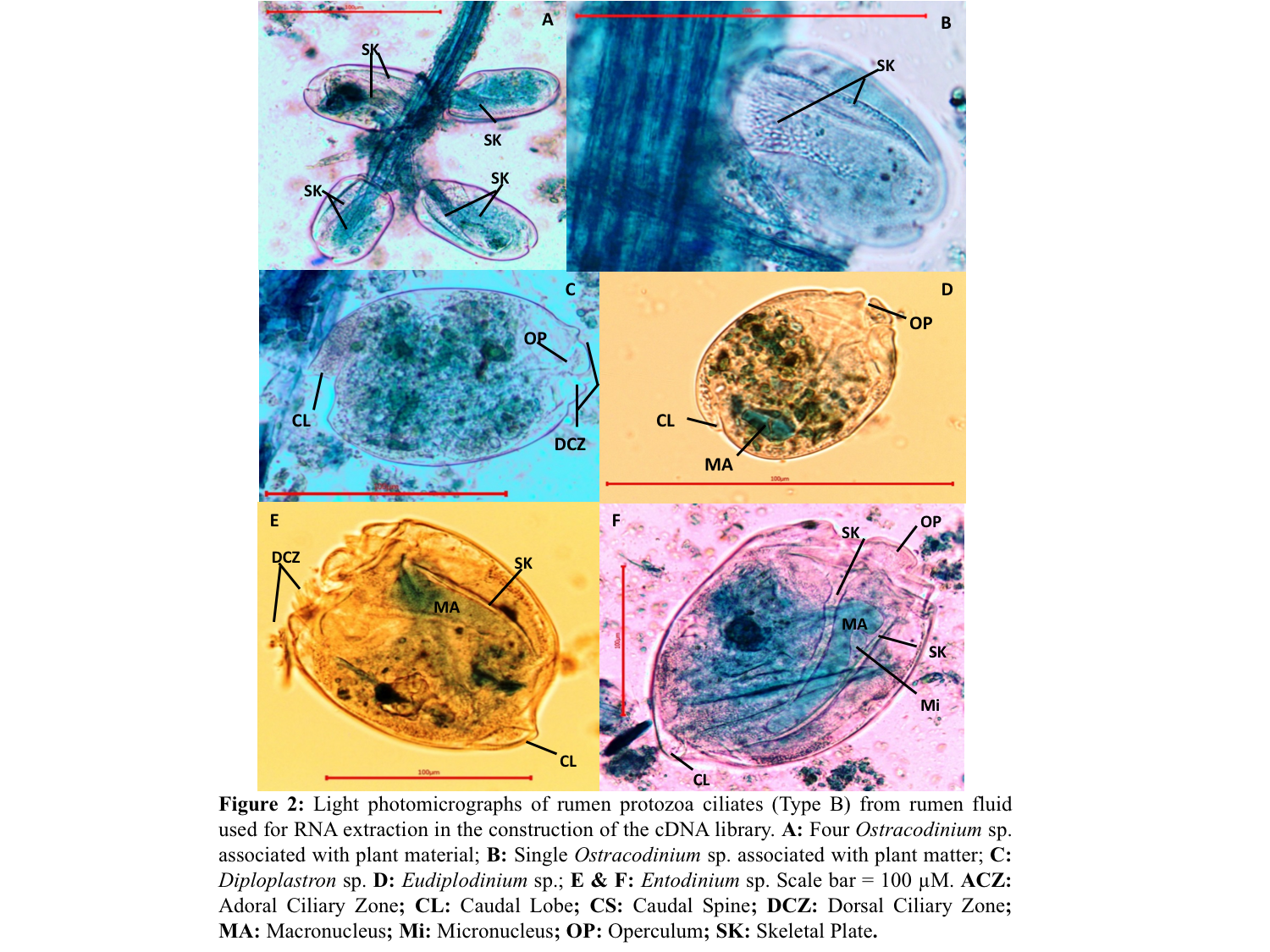

Supplement: Supplementary file 2 [file Image_2.tiff]

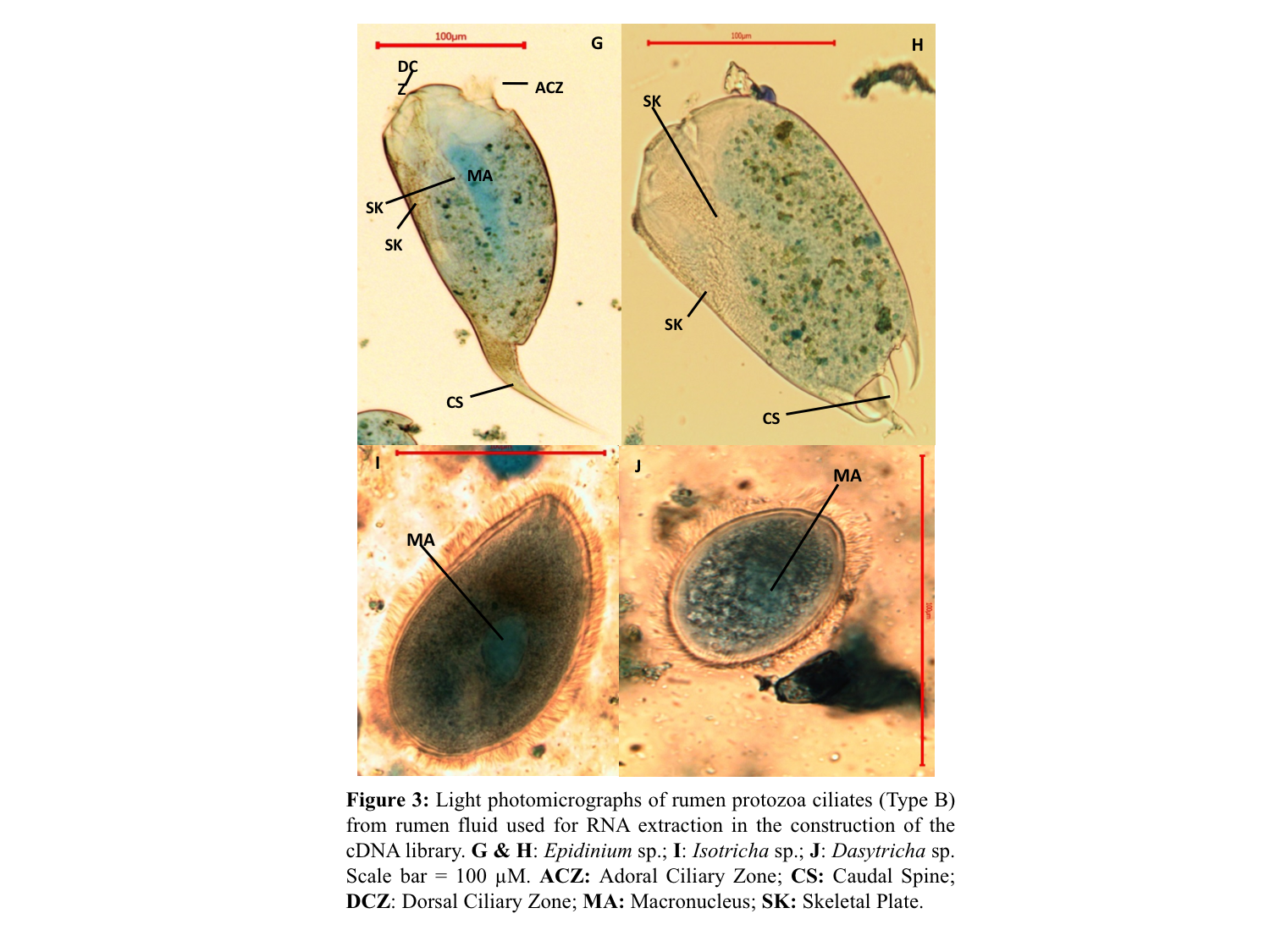

Supplement: Supplementary file 3 [file Image_3.tiff]
